# Scale Dependent Behavioral Responses to Human Development by a Large Predator, the Puma

**DOI:** 10.1371/journal.pone.0060590

**Published:** 2013-04-17

**Authors:** Christopher C. Wilmers, Yiwei Wang, Barry Nickel, Paul Houghtaling, Yasaman Shakeri, Maximilian L. Allen, Joe Kermish-Wells, Veronica Yovovich, Terrie Williams

**Affiliations:** 1 Environmental Studies Department, Center for Integrated Spatial Research, University of California Santa Cruz, Santa Cruz, California, United States of America; 2 School of Biological Sciences, Victoria University, Wellington, New Zealand; 3 Ecology and Evolutionary Biology Department, University of California Santa Cruz, Santa Cruz, California, United States of America; University of Western Ontario, Canada

## Abstract

The spatial scale at which organisms respond to human activity can affect both ecological function and conservation planning. Yet little is known regarding the spatial scale at which distinct behaviors related to reproduction and survival are impacted by human interference. Here we provide a novel approach to estimating the spatial scale at which a top predator, the puma (*Puma concolor*), responds to human development when it is moving, feeding, communicating, and denning. We find that reproductive behaviors (communication and denning) require at least a 4× larger buffer from human development than non-reproductive behaviors (movement and feeding). In addition, pumas give a wider berth to types of human development that provide a more consistent source of human interference (neighborhoods) than they do to those in which human presence is more intermittent (arterial roads with speeds >35 mph). Neighborhoods were a deterrent to pumas regardless of behavior, while arterial roads only deterred pumas when they were communicating and denning. Female pumas were less deterred by human development than males, but they showed larger variation in their responses overall. Our behaviorally explicit approach to modeling animal response to human activity can be used as a novel tool to assess habitat quality, identify wildlife corridors, and mitigate human-wildlife conflict.

## Introduction

Understanding the spatial scale of organismal response to human development is critical to unifying basic and applied ecology. How animals respond to human activity can influence population and community dynamics [1], [2], serve as the basis for designing wildlife reserves and corridors [3], and inform the mitigation of human-wildlife conflict [4], [5]. This is particularly true for large carnivores because of their vast home range requirements, disproportionate impacts on community composition [6], and central role in reserve design efforts [3].

Among mammalian carnivores, declining habitat fragment size leads to a predictable loss order of species according to body size and other ecological characteristics [7]. Large predator avoidance of smaller habitat fragments leads to mesopredator release - an increase in smaller predators and a consequent decrease in small mammals and birds [2]. While the pattern of human development can be to parcelize habitat into distinct fragments of varying size, human development (particularly in early stages) often consists of lines and blocks of developed areas that do not completely circumscribe natural areas, but instead create a variegated mosaic of natural and developed land [8]. In such variegated landscapes, the scales at which important animal behaviors related to survival and reproduction become impacted by human development will likely influence patterns of ecological function and determine the efficacy of conservation planning.

Perceptions of risk often shape landscape use by animals [9]. Individuals must balance the potential rewards gained from attaining food with the potential cost of being killed or displaced by a predator. This holds true for large carnivores as well [10], [11], who often avoid humans - a major source of mortality. The scale at which large carnivores avoid humans is likely to be influenced by the type of behavior under consideration. Evolutionary theory predicts that the strength of selection on a behavior will correlate with the fitness consequences of that behavior [12]. If a large carnivore is displaced from its kill near humans, it might lose a meal, but the same individual displaced from an established communication post or den site risks losing mating opportunities or offspring. As such, we predict that reproductive behaviors will have undergone stronger selection pressure than non-reproductive behaviors, thus requiring a larger buffer from human development than non-reproductive behaviors. Furthermore, we predict that types of human development that provide more varied sources of human interference will be given a wider berth (i.e. residences will be more strongly avoided than arterial roads). Around residences human activity can include car traffic, people walking and talking, dogs barking, nighttime lighting and many other potential activities that might negatively impact pumas. In contrast, arterial roads in our area are largely limited to intermittent car traffic.

Here we provide a novel approach to determining the spatial scale at which a large carnivore, the puma (*Puma concolor*), responds to human development when it is moving, feeding, communicating, and denning. We test whether the scale of response is different for reproductive (communicating and denning) versus non-reproductive behaviors (moving and feeding) and discuss how such analyses might be used as the basis for evaluating habitat quality, identifying wildlife reserves and corridors, and mitigating human-wildlife conflict. While our focus is on the puma, our approach should generalize to many other carnivore species and possibly other taxa as well.

## Methods

### Ethics Statement

The puma capturing, handling and monitoring for this research were reviewed by the Animal Care and Use Committee at the University of California, Santa Cruz (protocol #Wilmc1101). Approval for capturing, handling and taking samples from pumas was granted by the California Department of Fish and Game.

### Study Area

**Figure 1:**
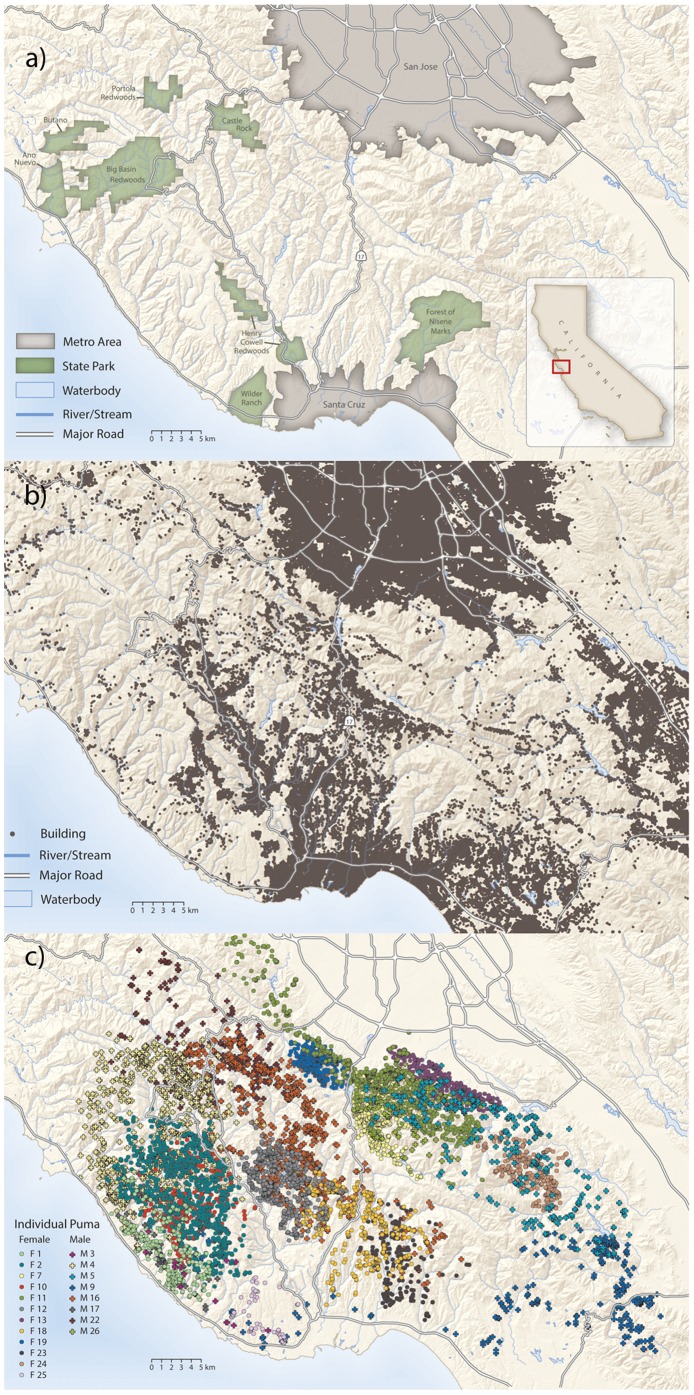
a) Map of the study area showing b) digitized housing locations and c) 4-hour movement locations of 20 pumas. Circles indicate females and pluses indicate males.

We conducted our study in the Santa Cruz Mountains of California (Fig. 1a). The mountain range is bounded by the city of San Francisco to the north, several urban municipalities to the east, mixed farmland and residential development to the south, and the Pacific Ocean to the west. Our 17,000 km^2^ study area overlaps Santa Cruz, San Mateo, and Santa Clara counties with elevation ranging from sea level to 1155 m. Vegetation communities on the west side of the mountains are dominated by redwood (*Sequoia sempervirens*) and douglas fir (*Pseudotsuga menziesii*) at low elevations, with patches of grassland, live oak (*Quercus* spp.), and coastal scrub immediately adjacent to the ocean. At higher elevations, mixed oak, conifer, and madrone (*Arbutus menziesii*) forests predominate. The land on the east side of the mountains is hotter and drier, characterized by shrub communities on ridges and south facing slopes, and mixed oak and bay laurel (*Umbellularia californica*) on north facing slopes and valleys. The climate is Mediterranean with the majority of annual precipitation occurring between November and April. Average annual precipitation varies from 58 cm to 121 cm throughout the mountain range.

Land use within the study area is varied as well. Large state and county parks and privately-held properties create a mosaic of relatively vast and intact areas of native vegetation. These large properties are bisected by varying amounts of development, from urban to rural. A major freeway (Highway 17) divides the study area. Agricultural activity is limited, with small vineyards, vegetable farms, and ranching operations (varying from a few goats to a few hundred cattle) interspersed throughout the mountains and along the coast.

### Puma Capture and Collaring

Pumas were captured from 2008–2011 using trailing hounds, cage traps, or leg-hold snares and anesthetized with Telazol (Fort Dodge Laboratories, Fort Dodge, IA, USA). Once anesthetized, pumas were sexed, weighed, measured, fit with an ear tag, and collared using a combined GPS/radio telemetry collar (Model GPS Plus 1D, Vectronics Aerospace, Berlin, Germany). Collars were programmed to take a GPS fix every 4 hours and had a mean (± se) fix rate of 86% (±1%). Data were remotely downloaded from the collar every 4 weeks via UHF or, alternatively, transmitted via cell phone towers every 1–3 days depending on collar configuration. Predicted battery life varied from 1–2 years depending on battery size. We attempted to recapture all pumas whose batteries failed prematurely or were scheduled to run out and fit them with new collars and/or batteries.

### Behavior Determination

Previous studies on pumas have explored behavior time budgets [13], minimum area requirements [14], dispersal of juveniles in fragmented habitat [15], and the relationship between urban development and puma presence [16], [17]. We expand upon these studies by using GPS data combined with extensive field reconnaissance to determine the spatial location of pumas while exhibiting four behaviors chosen to reflect both reproductive and non-reproductive activities: feeding, moving, communicating, and denning, and how these are influenced by natural and anthropogenic factors.

#### Feeding

We defined feeding sites as locations where pumas were likely to have killed a large prey, typically blacktail deer (Odcoileus hemionus columbianus). North American pumas are a generalist predator but are heavily dependent on a diet of large ungulates to survive and reproduce. In the Santa Cruz Mountains, pumas kill deer roughly once a week. Previous work found that GPS data can be used to accurately predict the locations of large prey, defined as mammals >8 kg, but not of small prey [18]; thus we chose to restrict our definition of a feeding site to places where pumas killed large prey. To find these locations, we classified potential feeding sites by generating GPS position clusters using a custom program integrated in the Geographical Information Systems program ArcGIS (v.10; ESRI, 2010) using the programming languages R (v.2.1.3.1; R Development Core Team, 2010) and Python (v. 2.6; Python Software Foundation, 2010). We adapted the algorithm developed by Knopff et al. [18] and identified possible feeding sites when two points occurred within 100 meters and six days of one another. We then calculated the geometric center between those two points. We expanded the cluster and recalculated the center if an additional point was located within 100 meters from the center and temporally separated from one of the original points by no more than six days. We iterated this process until no more points fit that criteria and moved on to the next potential kill site. After a cluster was identified, the program determined several descriptive characteristics of the cluster including: the coordinates for the geometric center of all points, the total amount of time between the first and last point of the cluster, the total number of points in the cluster, the number of night points in the cluster, the ratio of night to total points, the ratio of points in the cluster to points not in the cluster during the same time frame, a binary variable identifying whether the cluster lasted for more than 24 hours (1) or not (0), the number of cluster points adjusted for the success rate of the location fixes, and the fidelity to the cluster (number of points in the cluster subtracted by points away from the cluster). For each cluster, we also calculated the minimum, maximum, and average distances of all points from the center.

We visited 224 potential feeding sites identified by the clustering algorithm. We investigated the sites in reverse chronological order from the date the data were downloaded. We attempted to visit clusters within two weeks of the puma leaving the site, but occasionally waited longer because of logistical constraints. We programmed the geometric centers of each cluster into handheld GPS devices we carried into the field. Before proceeding to the cluster, we examined the spatial configuration of the points to determine whether we noticed any clumping of cluster points. If there was additional grouping within a kill site, we visually estimated the centers and also programmed them into our GPS devices. We began our searches at the geometric centers of all clusters and spiraled outwards until we reached the maximum distance a point was recorded from the center. We searched each cluster for at least 30 minutes. If we located prey remains, we labeled it as a puma kill if the decomposition status matched the time-frame over which the cluster was generated. For each prey carcass we found, we determined the age class, gender and species.

Each cluster, *y_i_* for *i* = 1,…, 224, was coded as 1 if prey remains were found and 0 otherwise. We used a logistic regression model given by,
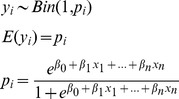
(1)to estimate the probability, *p*_i_, that a cluster was a kill using each of the cluster characteristics described above as predictor variables *x* with coefficients *β*. We selected the model with the lowest Akaike Information Criteria (AIC) [19] among all combinations of the predictor variables to estimate the probability that a cluster was a kill. We then used the intersection of specificity and sensitivity curves generated from the model as a break point in the probability [20]. Clusters with probability values above the break point were labeled as kills.

#### Movement

We defined movement locations as recorded GPS points not associated with a kill cluster or den site. Specifically, we removed all GPS points associated with a kill cluster (except for the first point to occur at the cluster) or den site from the GPS dataset. The remaining locations thus indicate periods when the pumas were likely moving or bedding.

#### Communication

Pumas communicate with each other through the use of scent markings called scrapes [21]. Scent marking is common among mammals and serves a dual purpose of advertising high competitive ability and attractiveness to the opposite sex [22]. Puma scrapes consist of a pile of leaves or duff that have been scraped together in a characteristic fashion on which they urinate [21]. The vast majority of scrapes are made by males, and are used to advertise their presence to conspecifics. Males will commonly scrape near trail junctions, ridges, kill sites, and on the sides of roads and trails. The same animals visit certain scrape locations that we call ‘community scrapes’ (defined as 3 or more scrapes within 9 meters squared of each other) repeatedly and mark adjacent to or on top of previous markings.

We located potential community scrapes using a modification of the custom program we developed for identifying kill sites, we located potential community scrape sites by finding clusters of male GPS locations where the male visited an area within 300 meters of a previous location two or more times, with visits separated by more than seven days. We then searched by foot all the trails, roads and ridge tops near the cluster. We recorded the actual GPS locations of each community scrape that we found with handheld GPS units. We also opportunistically recorded community scrapes in our extensive exploration of the study area while out investigating kill sites or trapping animals. Sites suggested by the GPS locations of returning males identified most of the community scrapes that we located. We maintained remote cameras at 44 (chosen so as to represent broad spatial coverage of the study area with different groups of pumas) of these sites for periods as long as 3 years, and in each case these were sites that were visited on a regular basis by territorial males. Females also visited community scrapes when looking for mates as evidenced by photographs of males and females together as well as GPS location data of males and females together.

#### Denning

Female pumas establish natal nurseries in thick vegetation or rock piles [21]. Females localize in these spots for a number of days after parturition and continue to return to the site for up to a few weeks until their offspring are mobile enough to change locations. We found dens by investigating female GPS data for clusters of points persisting for a week or more to which females made repeated return visits. In one case, the GPS unit on the puma’s collar failed and so we located her den site by triangulating her location using VHF telemetry multiple times over several days. We then investigated these clusters between 4 to 6 weeks of the female first localizing there and recorded the location of where we found the kittens using a handheld GPS.

### Statistical Analysis

We used resource selection functions (RSFs) under a use-availability design [23] to evaluate the relative puma preference for different habitat variables under each of the four different behavior types. The use-availability design generates relative probabilities of use by calculating the use of a habitat or environmental covariate relative to that covariate’s availability to the animal. Preference, *w*, for environmental covariate, *x,* with coefficient, *β,* is often estimated using the exponential model,
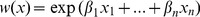
(2)
[24]. Different animals might respond differently to similar availabilities of environmental covariates, however. If this is the case, correct population-level inferences need to account for among individual differences [25]. This can be accomplished by adding a random effect which models variability among *k* pumas in their response to different covariates. The resulting mixed effects model thus allows for both conditional inferences about individual animals as well as marginal inferences about the population and correctly accounts for different sample sizes among individuals [26]. The model with interaction terms for the vector **x** of *I* covariates is given by,
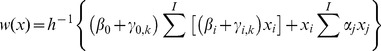
(3)for *k* pumas where the *β*’s are fixed coefficients, *γ*’s are normal random coefficients with mean 0 and covariance *Ω*, *α*’s are fixed coefficients for interaction terms, and *h* is a log-linear link. We added an intercept term to the mixed effects model to allow for variation among pumas in sample size and selection probability [26]. We fit the model using the lmer package in R with a binomial link, which has been shown to provide accurate parameter estimates for log-linear models [27].

While variation in use patterns can be attributed to differences in behavior among individuals, they can also be caused by differences in the overall availability of habitat covariates among individuals [25], [28]. As such, we tested for a functional response in puma use of areas in relation to housing density by regressing the random coefficients from equation 3 corresponding to housing density against the mean level of housing density that each puma experienced.

We calculated ‘use’ locations (coded as a 1) as follows for each behavior: we coded movement ‘use’ as each GPS location not associated with a feeding cluster or den site, feeding ‘use’ as the center of each predicted kill cluster, communication ‘use’ as the location of each community scrape, and denning ‘use’ as the location of each den site.

We generated ‘available’ locations (coded as 0) by choosing random points from the movement paths of each puma. Specifically, we used a matched-case control design whereby for each movement ‘use’ location, we calculated 5 random ‘available’ locations by adding 5 random vectors to the previous 4-hour location. The angle of these vectors was chosen from a uniform (0,2π) distribution, while the magnitude was sampled with replacement from a vector representing all the 4-hour movement distances, not associated with kill clusters, that the puma moved to during the period for which we collected data on it. In this way, we could then compare the actual location that a puma visited with 5 locations to which it could have visited. Data for which there was no GPS location 4 hours prior were discarded. Available locations for each behavior were then randomly sampled from this dataset such that a 5∶1 ratio of ‘available’ to ‘use’ points was maintained for each behavior.

For the communication model, the sampling regime adopted to record ‘use’ locations resulted in some observations that were highly clustered in space and recorded in close proximity to one another. Data collected in this manner introduces spatial structure that can have detrimental effects on statistical inference and severely confound parameter estimates from regression models [29]–[31]. As such, to explicitly account for any latent spatial effects in the analysis, we adopted a spatial filtering framework based on spatial eigenvector mapping (SEVM) [32]. Also, because we could not definitively assign scrape locations to particular individuals, we conducted our RSF of communication at the population level only (*γ* = 0, eq. 3).

SEVM captures latent spatial structure in a dataset as a set of eigenvectors extracted from a connectivity matrix expressing spatial relationships among spatial units. Each eigenvector represents a synthetic covariate whose linear combination with other eigenvectors constitute a spatial filter, or a set of proxy variables that remove the inherent spatial structure from regression models by treating this spatial structure as a missing variable. In our implementation, we generated eigenvectors using Moran’s Eigenvector Maps [33], [34] based on a binary spatial weighting matrix with neighbor relationships constructed by connecting all points within a fixed distance threshold. To give more weight to short-distance effects, we defined the truncation distance using the intercept of spatial correlograms for residuals from spatially naive models. Eigenvectors were selected by adding each set to the model until the spatial autocorrelation of residuals, measured using a permutation based global Moran’s *I*, were below a minimum desirable level (*p*<0.05). In this approach, we retained the eigenvector(s) best reducing spatial structure in our model and included them, along with other covariates, as predictors in the final specification. Calculations were performed using a modification of the spdep package in R [35].

#### Covariates

We included land cover, topographic, and fragmentation covariates as predictor variables in our models. All covariates were in a raster format at a nominal spatial resolution of 30 meters. We divided land cover into grassland, shrub, forest, and wetland cover types (US Geological Survey, Gap Analysis Program (GAP). May 2011. National Land Cover, Version 2). Pixels identified as agriculture were almost entirely rangeland. As such we collapsed these into our grassland cover type. For each pixel we also calculated its elevation, slope, aspect, and distance to the closest perennial water source.

Our human development covariates were derived from housing structures and roads. The location of each house or structure in the study area was digitized manually from high-resolution satellite imagery for rural areas, and calculated directly from a street address layer provided by the counties for urban areas (Fig. 1b). The reason for using two methods for digitizing house locations is that street address layers essentially provide the location of every mailbox, which for urban areas is typically quite close to the location of the house, while for rural areas might be up to a few kilometers away from the actual residence. The housing density of each 30 m×30 m pixel for our study area was then calculated from the housing points by applying a bivariate radially-symmetric Epanechnikov kernel with a scale parameter *h* to the location of each house and then summing the resulting densities at each 30 meter cell. The choice of *h* determines the width of the kernel and hence the relative strength of the housing impact as you move away from each house. At the landscape scale, large values of *h* result in housing density maps where the influence of human structures extends far into undeveloped land with a shallow slope. Small values of *h*, conversely, result in housing density maps where human influence falls off sharply when transitioning from human structures to undeveloped land. Consequently, different values of *h* will strongly influence the inference made about the impact of housing density on puma behavior. As such, we incorporated housing densities derived from multiple values of *h* varying from 10–2000 meters into our models and used model selection criteria to choose the best fitting *h* to the nearest 50 meters.

There are three broad categories of roads in our study area: arterial roads, which we define as roads with traffic speeds over 35 mph, neighborhood roads on which people live, and fire/logging roads which occur in undeveloped areas. Fire/logging roads are most likely an attractant to pumas because they facilitate travel, but we did not include these in our analysis because of the uneven accuracy of the data among properties. We also did not include neighborhood roads because these are highly correlated with housing locations such that our housing density layer captured the variance explained by neighborhood roads. For arterial roads, we included the distance to the nearest road of each location as a covariate.

For each behavior we fit models with multiple combinations of the predictor variables and chose the best models in each behavioral category as those that minimized the AIC. The scale of housing density which best fit data in each behavioral category was selected by finding the combination of covariates and housing density of scale *h* which minimized the AIC. We then evaluated our predictions by comparing housing density scales and slope coefficients (of the housing density and distance to arterial road variables) among behaviors. Due to the small sample size of den sites, we limited our analysis of this behavior to univariate fixed effect regressions of roads and housing density. Statistical models were fit using the lme4 package in R. All covariates were normalized 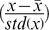 to improve model convergence and to facilitate comparison of model coefficients among covariates. We also made sure that no candidate models had covariates exhibiting high levels of colinearity (r>0.7).

In order to illustrate possible extensions of our results, we used the resultant behavioral maps to predict corridors crossing Highway 17, – the major freeway and possible barrier to movement in our study area. We selected the largest patches where communication and denning are unimpeded by housing density on either side of Highway 17. We then inverted and re-scaled relative use probabilities from our movement model to create a travel cost layer for pumas, which we used to calculate corridors of least-cost paths (using the Corridor function in ArcGIS 10.0) across Highway 17 between core areas.

## Results

Here we report the results of data collected on 20 pumas (12 females, 8 males, see Fig. 1c for movement data). One of the males dispersed during the study period while the rest of the animals were residents. The mean (± se) number of days that we recorded location data from each puma was 203 (±28) days. We recorded 21,053 locations for a mean (± se) of 1052 (±166) locations per animal. We located 183 community scrape locations and 10 den sites from as many different females. Community scrape locations were distributed both near and far from male territorial boundaries.

We visited 224 GPS clusters and located prey remains at 115 of these. The model that best predicted (ΔAIC = 0) whether a GPS cluster was a kill site included terms (coef, SE) for an intercept (−2.06, 0.42), binary variable identifying whether the cluster lasted for more than 24 hours (1.16, 0.41), fidelity to the cluster (0.05, 0.02), ratio of number of night points to total points (1.66, 0.56) and total number of night points (0.10, 0.07). The ability of the model to discriminate between kills and non-kills was ‘excellent’ as determined by an area of 0.80 under the receiver operator curve (ROC) [20]. Specificity (probability of correctly classifying a kill as a kill) and sensitivity (probability of correctly classifying a non-kill as a non-kill) curves intersected at a probability cut off of 0.485. This yielded a sensitivity of 70% and a specificity of 78%. At this cut off the algorithm predicted 105 kills. Applied to the entire GPS dataset, the algorithm predicted 667 kills.

**Figure 2:**
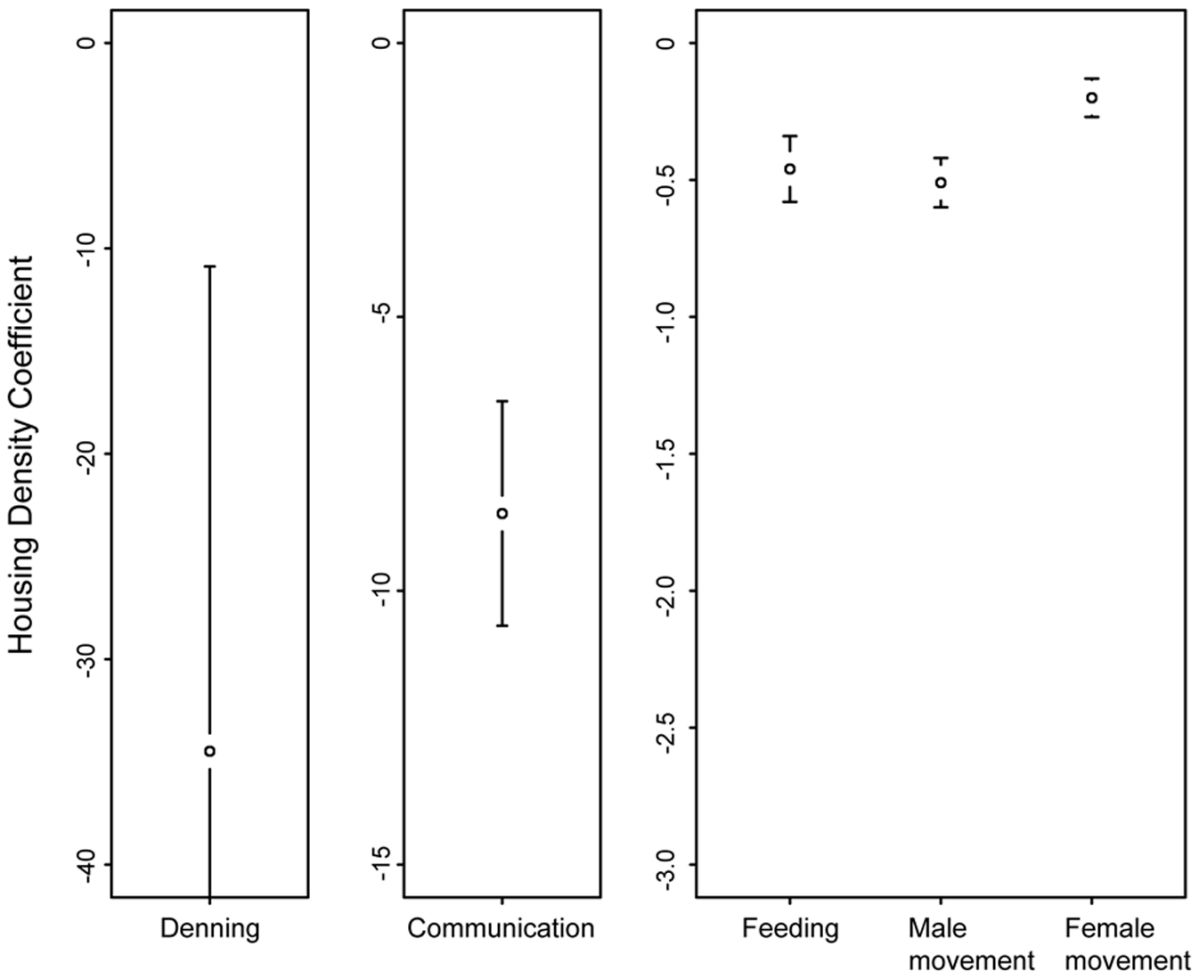
Relative sensitivities of each behavior to housing density as indicated by the fixed regression coefficient (± s.e.) of housing density in each behavioral model. These represent the relative log odds of pumas exhibiting each behavior for a unit change in normalized housing density holding other covariates constant. Note the difference in the y-axis scale among panels.

**Figure 3:**
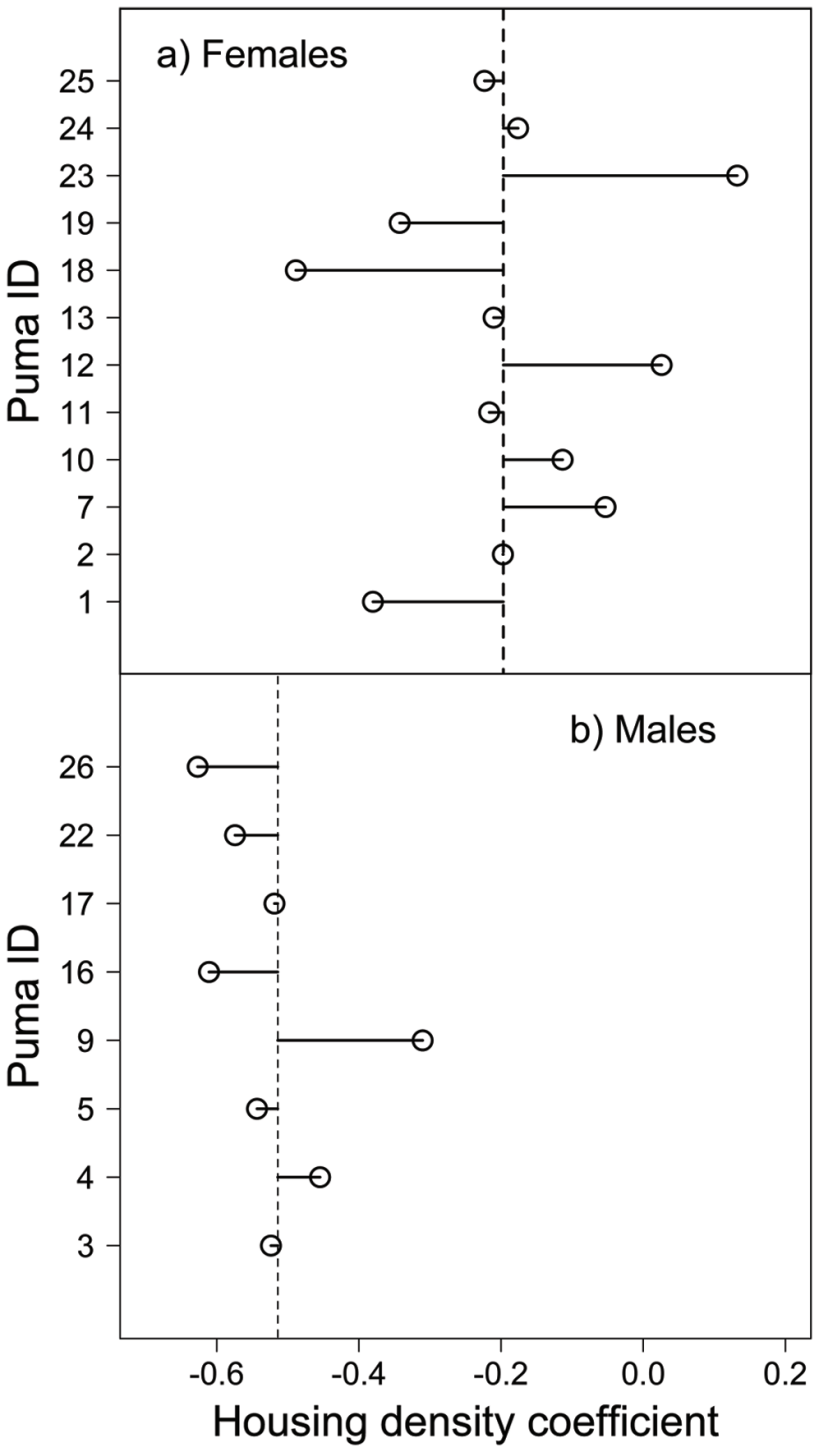
Estimates of the random effect coefficient of housing density for each puma are plotted in relation to the marginal prediction (dashed line) for a) females and b) males. Females were more tolerant of human development and displayed greater variation in their response than males, likely due to life history differences related to breeding and raising young. The sole dispersing male (M9) in our sample exhibited a tolerance of housing density more similar to that of females than other males.

The RSF analysis revealed that feeding, movement, communication, and denning were best fit by models with a housing density scale parameter, *h*, of 50 m, 150 m, 600 m and 600 m respectively (Table 1). These scales were not sensitive to which covariates were in the model (see supplemental information Table S1). At these scales, plots of the population level response of the relative probability of use in relation to housing density reveal an increasing negative slope as behavior changes from movement and feeding to communication and denning (Fig. 2). Housing density scale, *h*, influenced the magnitude of these coefficients, but comparison among behaviors at different scales still revealed strong differences (see supplemental information Table S1). The movement of pumas in response to housing density was variable, with males displaying a stronger aversion to houses than females (Fig. 3). Interaction terms also revealed that pumas were less deterred in their movement by housing when slopes were steep and more deterred by housing when near water. Arterial roads did not significantly influence puma movement, and were a small attractant to puma kill site selection (*β*_road_ = −0.13). Conversely, pumas selected against arterial roads when communicating (*β*_road_ = 0.40), especially in flat areas, and when denning (*β*_road_ = 1.7). The response of pumas to natural environmental covariates is detailed in table 1.

**Table 1:** The best fit RSF model for each puma behavioral category (in italics).

	Fixed effects	Coefficient	Std. error
***Feeding***	gender	0.11	0.11
***h*** ** = 50** **m**	elevation	−0.60	0.11
	distance to road	−0.13	0.05
	grassland	−0.59	0.27
	housing density	−0.46	0.12
	gender x elevation	0.57	0.12
	**Fixed effects**	**Coefficient**	**Std. error**
***Movement***	gender	0.02	0.02
***h*** ** = 150** **m**	slope	−0.09	0.03
	elevation	−0.05	0.01
	distance to water	0.04	0.01
	grassland	−0.35	0.14
	forest	0.30	0.05
	shrub	0.30	0.05
	housing density	−0.51	0.09
	gender x housing density	0.32	0.11
	slope x housing density	0.15	0.03
	elevation x water	−0.05	0.01
	water x housing density	−0.09	0.03
	**Random effects**	**Variance**	**Correlation**
	housing density	0.03	
	slope	0.01	0.09
	**Fixed effects**	**Coefficient**	**Std. error**
***Communication***	housing density	−8.59	2.05
***h*** ** = 600** **m**	distance to road	0.40	0.15
	slope	−1.35	0.19
	road x slope	−0.85	0.19
	**Fixed effects** *	**Coefficient**	**Std. error**
***Denning***	housing density	−34.48	23.6
***h*** ** = 600** **m**	distance to road	1.70	0.52

* Coefficients estimated independently using univariate regressions due to low sample size.

Sample sizes for each analysis were as follows: Feeding –20 pumas, 667 kill sites; Movement –20 pumas, 21,053 movement locations; Communication –183 community scrapes; Denning –10 nurseries.

The scale, *h*, of housing density in the best-fit model is reported below each of the listed behaviors.

**Figure 4:**
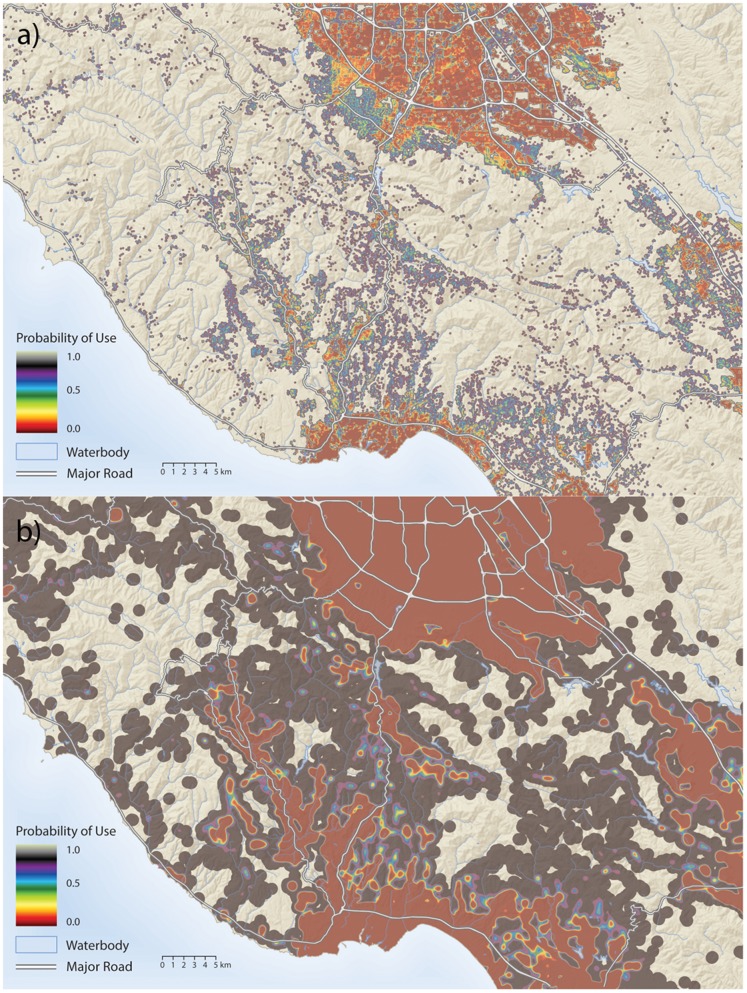
Maps of the study area displaying the relative probability of use by pumas in relation to housing density when they are either a) feeding, or b) communicating. Intermediate colors on the feeding map are areas that pumas are likely to avoid, but will still visit occasionally to hunt and feed. It is here that most puma mortality occurs over conflicts with livestock owners. The light colored areas on the communication map represent areas where pumas are not impacted by housing density when communicating (or denning). These areas would make good candidates for reserves.

**Figure 5:**
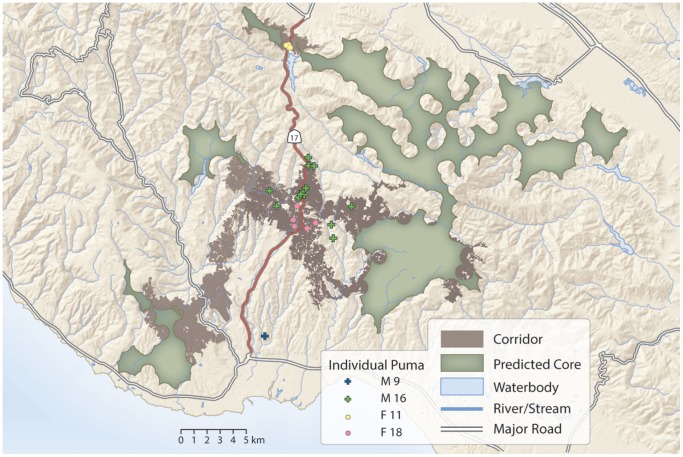
Map illustrating the top 0.5% least-cost corridors crossing Highway 17– the major freeway and barrier to movement in our study area. Predicted core areas on either side of the Highway 17 are those areas where reproductive behaviors are unimpeded by housing density. Corridors were generated using the puma movement model. For each consecutive pair of individual puma locations that bisect Highway 17 (representing crossing locations), we plot the location closest to the freeway.

The final model for each behavioral category was used to generate spatial predictions of habitat use in relation to housing density for feeding (Fig. 4a), and communication (Fig. 4b). The mapped predictions reveal large-scale spatial differences between habitat appropriate for feeding and communication in relation to housing density. Maps of movement were similar to feeding, while maps of denning were similar to communication and are not displayed here. Corridor predictions based on the reproduction and movement maps correspond closely to where pumas have been documented to cross Highway 17 (Fig. 5).

## Discussion

Our study examining the differential effects of human development on puma behavior revealed that pumas required a larger buffer from human development when exhibiting reproductive behaviors than non-reproductive behaviors. This is likely due to the fact that disrupting reproductive behaviors imparts a higher evolutionary cost than disputing feeding behaviors. As solitary animals with large home ranges, pumas use chemical and auditory cues to locate mates. These types of communication, however, are vulnerable to disruption by humans. Hikers, bikers and dogs can easily disturb chemical communication at scrape sites, which are often the first point of contact between males and females. Loud mating calls might also expose pumas to greater risk of disturbance or mortality from humans and/or dogs.

Previous work on puma movement has shown that pumas avoid residential development and 2-lane roads [16], [17], [36], [37]. Furthermore, when they do move close to human dominated areas, their traveling speeds increase [37]. Interaction terms from our movement model indicated that pumas were more likely to use areas near houses when traveling on steep slopes and less likely to utilize areas near houses when close to water. These interactions likely reflect the pattern that human activity tends to increase near water sources and decrease on steep slopes. Pumas were also more deterred by houses than by arterial roads, indicating that they require a wider berth from more predictable sources of human interference. In fact, pumas only avoided arterial roads when they were engaged in reproductive activities and displayed no aversion when moving or feeding.

There was substantial individual variation in puma response to housing density. Females were less deterred by housing density than males and showed larger variation in their response overall. Similar to results by Kertson et al [16] and Burdett et al [17], neither sex displayed a functional response to housing density, indicating that tolerance of housing was not a function of its overall availability in their home range. Though we were not able to explain differences among males and females, we suspect that they are due to life history differences. Females caring for dependent young, especially large cubs with high energetic demands, are more likely to be food-limited than males, and might be attracted to neighborhoods where prey are more abundant. Males, conversely, are highly repetitive in their movements, moving between communication sites and seeking out females. Since communication sites are remote, this would lead to a lower variation in overall male landscape use in relation to human development. The one male that did show ‘female-like’ tolerance for human development was a dispersing animal, a life history stage during which pumas are known to exhibit tolerance for more developed areas as they seek out their own territories [15]. Thus it is likely that those animals most likely to take on the higher risk associated with developed areas are young pumas and females with large cubs. While individual pumas showed differential responses to housing density, they might also respond differently to the configuration of residential development. For instance development along a linear boundary might induce a different response than development that circumscribes habitat. Future work is thus needed to disentangle potential interactions between the density and configuration of residential development.

Pumas in our study responded to natural environmental covariates in a similar fashion as reported elsewhere [17]. They were generally attracted to shrub, forest and water, which provide good hunting and escape habitat, and were deterred by grassland [but see 38], which lacks effective stalking cover. We did not include a covariate for the spatial distribution of prey density because heavy forest cover, high housing densities, and rugged terrain precluded accurate estimation of this covariate. We note, however, that deer, the primary prey of pumas in our study area, are known to be attracted to areas with higher housing density where they forage on high nutrient-irrigated gardens and landscaping [39]. As such, any bias induced in our model by not including prey density as a covariate would likely lead us to under predict the negative impact of human development on puma behavior.

Accurately measuring habitat quality is crucial to a variety of conservation and management issues. Recent work by Mosser et al. [40] using a 40-year spatially explicit dataset on African lion (*Panthera leo*) reproductive success and density revealed that areas of high reproductive activity were a better predictor of habitat quality than lion density, and that some areas of high lion density were actually population sinks. Common approaches to measuring habitat quality (such as RSF’s of GPS location data), however, assume a direct positive relationship between population density and habitat quality. This can be problematic if some areas of high density are actually population sinks. Long-term datasets on reproductive success are exceedingly rare, however, while GPS data sets are increasingly common. Assuming that the spatial dependency of reproductive success correlates with reproductive behavior, the behaviorally explicit approach we have demonstrated here can be used to assess habitat quality by conducting an RSF on the spatial response of reproductive behaviors to human development and environmental covariates. While this assumption remains to be tested, it holds promise for improving rapid assessments of habitat quality using GPS location data combined with strategic field measurements.

An emerging goal among conservation practitioners is to identify movement corridors among patches of high quality habitat so as to ensure metapopulation persistence and allow for species range shifts under climate change [41]. Quantitative methods used to assess connectivity and define corridors such as least-cost modeling [42] and circuit theory [43] require the identification of both habitat patches and resistance landscapes. These are often determined using existing protected areas and expert opinion, respectively [44]. Our approach, conversely, explicitly defines these areas, whether they are currently preserved or not, using real data. Spatial predictions of reproductive behaviors can be used to identify core areas, while spatial distributions of relative movement probabilities can be inverted to define a resistance landscape. As illustrated in figure 5, the corridors we estimated using this approach accurately capture where pumas are crossing highway 17.

Areas of high human-wildlife conflict, where animals threaten livestock, crops or human safety, can be a significant source of animal mortality, and can help shape people’s attitudes towards conservation [45]. This is evident from the mortality statistics for pumas in our study. Eight out of eleven adult puma mortalities to date were the result of depredations after pumas attacked domestic livestock. Similarly, Burdett et al [17] showed that pumas that selected for habitat nearer to humans had higher rates of mortality. Kertson et al [16] suggested that a threshold residential density exists at which puma-human interactions are likely to be maximized. Areas of intermediate feeding probability illustrated in Fig. 4a can serve as a predictive map of where such human puma conflict is most likely to occur. These are areas where pumas are moderately deterred by human development, but to which they will still make occasional foraging visits. It is during these visits that pumas are most likely to kill livestock (often pet goats in our area), and then be lethally removed by landowners for doing so. By identifying areas where conflict is most likely to occur, our model can help target education efforts designed to help landowners reduce losses of livestock to large carnivores, and consequently, reduce an important source of human-mediated large carnivore mortality.

The initial response by animals to anthropogenic changes in the environment is often behavioral [46]. By distinguishing the spatial response of distinct behaviors relevant to survival and reproduction in wild animals to human development, we are able to glean insights as to how animals are likely to respond to increased fragmentation in the environment and to identify strategic conservation regions. As new communities are planned, our analytical approach allows us to predict whether target species will cease communicating and denning, where human-wildlife conflicts are likely to take place, and how animals will use the landscape to move from one breeding area to the next. This approach can also be used to mitigate the effects of increased human development by informing the location of wildlife reserves, corridors and education efforts.

## Supporting Information

Table S1 **Influence of covariate combinations on the optimal housing density scale,** ***h*** **, for each behavior.**
(DOCX)

Table S2 **The effect of different housing density scales,** ***h*** **, on the housing density coefficient for each behavior.**
(DOCX)

Text S1 **To ensure that our statistical procedure for choosing the behaviorally specific scales at which pumas are responding to housing density was not an artifact of different covariates in the best fit model for each behavior, we computed AIC scores across scales of housing density ranging from 50–1000 meters for each behavior, using the covariates from the best fit model of each behavior reported in** **Table 1** **.**
(DOCX)
