# Supplementary material for: Scale Dependent Behavioral Responses to Human Development by a Large Predator, the Puma
Source: PLoS One. 2013 Apr 17;8(4):e60590. doi: 10.1371/journal.pone.0060590 (PMC3629074; doi:10.1371/journal.pone.0060590)
Supplement: Table S1 — Influence of covariate combinations on the optimal housing density scale, h , for each behavior. (DOCX) [file pone.0060590.s001.docx]

| **Table S1** – Checking the influence of covariate combinations on the optimal housing density scale, *h*, for each behavior. AIC scores are reported for multiple values of *h* for each behavioral model^*^ using the set of covariates from each of the best behavioral models reported in Table 1. The lowest AIC scores, indicating the best model, for each behavior and set of covariates are reported in bold face. | | | | |
| --- | --- | --- | --- | --- |
|  | | Behavior (AIC scores) | | |
| Covariates | *h* | Communication | Feeding | Movement |
| (From best fit communication model): housing density, distance to road, slope, road x slope | 50 | 512 | **3579** | 60089 |
|  | 100 | 503 | 3588 | 60000 |
|  | 150 | 498 | 3593 | **59993** |
|  | 200 | 492 | 3596 | 60004 |
|  | 400 | 482 | 3600 | 60072 |
|  | 600 | **462** | 3603 | 60143 |
|  | 800 | 484 | 3604 | 60201 |
|  | 1000 | 486 | 3605 | 60243 |
|  |  |  |  |  |
| (From best fit feeding model): gender, elevation, distance to road, grassland, housing density, gender x elevation | 50 | 597 | **3560** | 60179 |
|  | 100 | 638 | 3566 | 60089 |
|  | 150 | 632 | 3573 | **60080** |
|  | 200 | 629 | 3577 | 60087 |
|  | 400 | 608 | 3585 | 60147 |
|  | 600 | **593** | 3590 | 60214 |
|  | 800 | 597 | 3592 | 60271 |
|  | 1000 | 597 | 3593 | 60315 |
|  |  |  |  |  |
| (From best fit movement model): gender, slope, elevation, distance to water, grassland, forest, shrub, housing density, gender x housing dens, slope x housing density, elevation x water, water x housing density | 50 | 532 | **3586** | 59873 |
|  | 100 | 532 | 3592 | 59789 |
|  | 150 | 528 | 3593 | **59773** |
|  | 200 | 505 | 3592 | 59778 |
|  | 400 | 477 | 3595 | 59822 |
|  | 600 | **471** | 3597 | 59862 |
|  | 800 | 476 | 3597 | 59893 |
|  | 1000 | 477 | 3597 | 59918 |
| ^*^ We excluded denning behavior from this analysis due to low sample size. | | | | |
|  | | | | |
|  |  |  |  |  |
|  |  |  |  |  |
