# Supplementary material for: Scale Dependent Behavioral Responses to Human Development by a Large Predator, the Puma
Source: PLoS One. 2013 Apr 17;8(4):e60590. doi: 10.1371/journal.pone.0060590 (PMC3629074; doi:10.1371/journal.pone.0060590)
Supplement: Table S2 — The effect of different housing density scales, h , on the housing density coefficient for each behavior. (DOCX) [file pone.0060590.s002.docx]

| **Table S2** – The effect of different housing density scales, *h*, on the housing density coefficient for each behavior^*^, using the set of covariates from the best model for each behavior reported in Table 1. Coefficients from the best fit scale for each behavior were also reported in Table 1 and are indicated here in bold. | | | |
| --- | --- | --- | --- |
|  | Housing density coefficient | | |
| *h* | Communication | Feeding | Movement |
| 50 | -3.9 | **-0.45** | -0.41 |
| 150 | -5.37 | -0.36 | **-0.51** |
| 600 | **-8.59** | -0.11 | -0.28 |
| ^*^ We excluded denning behavior from this analysis due to low sample size. | | | |
