# Supplementary material for: Scale Dependent Behavioral Responses to Human Development by a Large Predator, the Puma
Source: PLoS One. 2013 Apr 17;8(4):e60590. doi: 10.1371/journal.pone.0060590 (PMC3629074; doi:10.1371/journal.pone.0060590)
Supplement: Text S1 — To ensure that our statistical procedure for choosing the behaviorally specific scales at which pumas are responding to housing density was not an artifact of different covariates in the best fit model for each behavior, we computed AIC scores across scales of housing density ranging from 50–1000 meters for each behavior, using the covariates from the best fit model of each behavior reported in Table 1 . (DOCX) [file pone.0060590.s003.docx]

**Text S1**

To ensure that our statistical procedure for choosing the behaviorally specific scales at which pumas are responding to housing density was not an artifact of different covariates in the best fit model for each behavior, we computed AIC scores across scales of housing density ranging from 50-1000 meters for each behavior, using the covariates from the best fit model of each behavior reported in Table 1. These results are reported in Table S1. Housing density scale was consistently 50m for feeding, 150m for movement and 600m for communication. We also investigated the influence of housing density scale, *h*, on the magnitude of the coefficient of housing density for each behavior by calculating the value of the coefficient at each of the scales predicted for each behavior (i.e. 50m for feeding, 150m for movement and 600m for communication). Not surprisingly, the results reported in Table S2 reveal that as the model fit declines within a behavior (by moving away from the best fit scale), the magnitude of the housing density coefficient declines. The relative strength of the coefficient across behaviors reveals, however, that housing density is still avoided much more strongly when animals are communicating, than when they are feeding or moving.
